# Repeated measures ANOVA and adjusted *F*-tests when sphericity is violated: which procedure is best?

**DOI:** 10.3389/fpsyg.2023.1192453

**Published:** 2023-08-30

**Authors:** María J. Blanca, Jaume Arnau, F. Javier García-Castro, Rafael Alarcón, Roser Bono

**Affiliations:** ^1^Department of Psychobiology and Behavioral Sciences Methodology, University of Malaga, Málaga, Spain; ^2^Department of Social Psychology and Quantitative Psychology, University of Barcelona, Barcelona, Spain; ^3^Department of Psychology, Universidad Loyola Andalucia, Seville, Spain; ^4^Institute of Neurosciences, University of Barcelona, Barcelona, Spain

**Keywords:** Greenhouse-Geisser adjustment, Huynh-Feldt adjustment, robustness, power, Mauchly test

## Abstract

**Introduction:**

One-way repeated measures ANOVA requires sphericity. Research indicates that violation of this assumption has an important impact on Type I error. Although more advanced alternative procedures exist, most classical texts recommend the use of adjusted *F*-tests, which are frequently employed because they are intuitive, easy to apply, and available in most statistical software. Adjusted *F*-tests differ in the procedure used to estimate the corrective factor *ε*, the most common being the Greenhouse-Geisser (*F-GG*) and Huynh-Feldt (*F-HF*) adjustments. Although numerous studies have analyzed the robustness of these procedures, the results are inconsistent, thus highlighting the need for further research.

**Methods:**

The aim of this simulation study was to analyze the performance of the *F*-statistic, *F-GG*, and *F-HF* in terms of Type I error and power in one-way designs with normal data under a variety of conditions that may be encountered in real research practice. Values of *ε* were fixed according to the Greenhouse–Geisser procedure ($\hat{\varepsilon }$). We manipulated the number of repeated measures (3, 4, and 6) and sample size (from 10 to 300), with $\hat{\varepsilon }$ values ranging from the lower to its upper limit.

**Results:**

Overall, the results showed that the *F*-statistic becomes more liberal as sphericity violation increases, whereas both *F-HF* and *F-GG* control Type I error; of the two, *F-GG* is more conservative, especially with large values of $\hat{\varepsilon }$ and small samples.

**Discussion:**

If different statistical conclusions follow from application of the two tests, we recommend using *F-GG* for $\hat{\varepsilon }$ values below 0.60, and *F-HF* for $\hat{\varepsilon }$ values equal to or above 0.60.

## Introduction

The general linear model (GLM) underpins the most widely used statistical procedures in the health and social sciences, namely analysis of variance (ANOVA), analysis of covariance, and linear regression, the general objective of which is to analyze how one or a set of independent variables affects or is related to a dependent variable. Blanca et al. (2018) found that parametric tests for means comparison and linear regression were the most commonly employed statistical procedures in this field, with a prevalence of 36.5 and 20.97% among published studies, respectively. Other authors have shown that ANOVA is one of the most widely used procedures in articles published in psychology journals (e.g., Kashy et al., 2009; Counsell and Harlow, 2017), with repeated measures ANOVA (RM-ANOVA) being more prevalent than ANOVA with a grouping factor (57.92% vs. 42.08%). Regarding the characteristics of these designs, Keselman et al. (1998) noted that 55.3% of studies reported a sample size of 60 or less, although the total sample size ranged from 6 to more than 1,000 participants. In the neuropsychological field, Goedert et al. (2013) found that the most common number of repeated measures was 2 or 3, followed by 4 or 6, with a mean total number of participants of 18.11 (median = 14.5).

RM-ANOVA uses the *F-*statistic to determine statistical significance, and this requires, among other assumptions, normality and sphericity. Previous Monte Carlo simulation studies have indicated that the Type I error and power of the *F*-test are not altered by the violation of normality when sphericity is fulfilled. For example, Blanca et al. (2023) carried out an extensive study, examining a wide variety of conditions that might be encountered in real research situations. They manipulated the number of repeated measures (3, 4, 6, and 8), sample size (from 10 to 300), and distribution shape (slight, moderate, and severe departure from the normal distribution), and considered both equal and unequal distributions in each repeated measure. The results showed that RM-ANOVA is robust under non-normality when the sphericity assumption is met with distributions having skewness and kurtosis values as large as 2.31 and 8, respectively, and also that empirical power did not decrease with the violations of normality tested in the study.

In one-way repeated measures designs, the sphericity assumption is met when the variances of the population difference scores for all pairs of treatment levels are homogeneous (Kirk, 2013) (for a definition in terms of the covariance matrix, see Kirk, 2013, pp. 306–310, and Langenberg et al., 2023). Sphericity is usually examined with the Mauchly test, whose null hypothesis states that the variances of the differences are equal. As recommended in some books (e.g., Gamst et al., 2008), applied researchers usually perform the Mauchly test as a preliminary analysis and then, depending on the results, decide on the subsequent analytic strategy. However, early simulation studies showed that the Mauchly test does not control Type I error under non-normality, and neither is it sensitive to small departures from sphericity (Huynh and Mandeville, 1979; Keselman et al., 1980), thus calling into question its usefulness as a preliminary test.

Several decades ago, Box (1954) showed that the Type I error rate of the *F*-statistic increases when sphericity is violated. This tendency toward liberality has been highlighted in several subsequent studies (Collier et al., 1967; Berkovits et al., 2000; Voelkle and McKnight, 2012; Haverkamp and Beauducel, 2017, 2019), which implies that use of the *F*-statistic may lead to the null hypothesis being rejected more often than it should. Box (1954) also pointed out that in a one-way RM-ANOVA, when sphericity holds, the *F*-statistic is distributed with (*K*-1) and (*K*-1) (*N*-1) degrees of freedom, where *K* is the number of repeated measures and *N* is the number of participants. However, when sphericity is violated, the *F*-statistic may follow another distribution, with the degrees of freedom reduced by the multiplicative factor epsilon (*ε*). Specifically, the *F*-statistic is distributed with *ε* (*K*-1) and *ε* (*K*-1) (*N*-1). The value of *ε* describes the degree to which sphericity is violated and it lies between its minimum bound, 1/*K*-1, and 1 (Geisser and Greenhouse, 1958). When sphericity holds, *ε* is equal or close to 1. The violation of sphericity is more severe the further the *ε* value is from 1 and the closer it moves toward its lower bound.

Adjusted *F*-tests have been proposed with the purpose of reducing the degrees of freedom associated with the *F*-statistic by the corrective factor *ε*. As the value of *ε* is unknown in the population, it must be estimated, and adjusted *F*-tests may be distinguished based on the estimator that is applied to the sample covariance matrix, the most common being the Greenhouse-Geisser (*F-GG*; Box, 1954; Geisser and Greenhouse, 1958; Greenhouse and Geisser, 1959) and Huynh-Feldt (*F-HF*; Huynh and Feldt, 1976) adjustments. These two estimators of *ε* are referred to, respectively, as $\hat{\varepsilon }$ and $\tilde{\varepsilon }$. According to Huynh and Feldt (1976), $\tilde{\varepsilon }$ ≥ $\hat{\varepsilon }$, although the two estimators are equal when *ε* reaches its lower bound. Huynh and Feldt (1976) also found that $\hat{\varepsilon }$ is a less biased estimator for low values of *ε*, whereas $\tilde{\varepsilon }$ is less biased for *ε* values above 0.75.

The use of adjusted *F*-tests is an alternative recommended in most classical texts (e.g., Maxwell and Delaney, 2004; Tabachnick and Fidell, 2007; Kirk, 2013) for when sphericity is violated. Although other more advanced procedures such as the linear mixed model have been developed, many researchers prefer to use adjusted *F*-tests because they are intuitive, easy to apply, and are available in most statistical data analysis software (e.g., IBM-SPSS, SAS, R, etc.). In the context of the present study, we conducted a preliminary search of Web of Science to gain a general idea of the prevalence of these tests in the recent scientific literature, focusing on the fields of psychology, behavioral sciences, psychiatry, social sciences, and educational research. The search identified around 3,000 articles published during 2021 and 2022, indicating the widespread use of adjusted *F*-tests in these fields. Although we did not perform an exhaustive systematic review, this preliminary search nevertheless shows the extent to which adjusted *F*-tests are applied in these key areas of research.

The performance of adjusted *F*-tests has mainly been studied with split-plot designs and with simultaneous violations of both normality and sphericity (Keselman and Keselman, 1990; Quintana and Maxwell, 1994; Keselman et al., 1995, 1999; Fernández et al., 2010). This means that there are no recent studies that extensively analyze the effect of the violation of sphericity, regardless of non-normality. Monte Carlo simulation studies with one-way designs are very scarce, and vary depending on the variables manipulated. Type I error is generally interpreted using Bradley’s (1978) liberal criterion, according to which a procedure is considered robust if the Type I error is between 2.5 and 7.5% at a significance level of 5%.

Berkovits et al. (2000) simulated data from designs with *K* = 4, small sample sizes (*N* = 10, 15, 30, and 60), and different values of *ε* (0.48, 0.57, 0.75, and 1). Their results showed that the *F*-statistic was liberal at lower *ε* values (0.48 and 0.57), regardless of the distribution shape and sample size. When normality was tenable, both *F-GG* and *F-HF* were robust in all sphericity conditions, with Type I error within Bradley’s limits. They concluded that when a violation of sphericity occurs alone, both procedures (*F-GG* and *F-HF*) offer reasonable control of Type I error. Similar results were obtained by Muller et al. (2007), who simulated normal data with 9 repeated measures, *ε* values of 0.28, 0.51, and 1, and small sample sizes (*N* = 10, 20, and 40). They found that *F-GG* and *F-HF* were robust to sphericity violations with adequate statistical power, leading them to recommend the use of these adjusted *F*-tests over more complex models, such as linear mixed models. Oberfeld and Franke (2013) included designs with *K* = 4, 8, and 16, different structures of the covariance matrices with *ε* equal to 0.50 and 1, and sample sizes between 3 and 100. The results with normal data indicated that *F-HF* was more robust than *F-GG*, especially with small sample sizes and a large number of repeated measures, insofar as *F-GG* was found to be conservative in some conditions. Haverkamp and Beauducel (2017) also recommended the use of *F-HF* over *F-GG* in these same conditions, focusing on designs with 3, 6, and 9 repeated measures, normal data, and sample sizes of 20, 40, 60, 80, and 100. The results of a subsequent study by the same authors (Haverkamp and Beauducel, 2019), in which they tested designs with 9 and 12 repeated measures and smaller sample sizes (15, 20, 25, and 30), likewise supported the use of *F-HF*, which offered correct control of Type I error when sphericity was violated.

The above findings contrast with those of other researchers and with what is indicated in some classical books. For example, Voelkle and McKnight (2012) found that although both adjusted *F*-tests controlled fairly well for Type I error, *F-GG* outperformed *F-HF*. Kirk (2013) and Maxwell and Delaney (2004) likewise proposed using *F-GG*, based on early studies showing that *F-HF* achieved less control over the Type I error rate. Other authors, drawing on the results of Huynh and Feldt (1976), even pointed out the possibility of using one strategy or the other depending on the expected value of *ε*, such that *F-GG* should be used if *ε* is believed to be less than 0.75, whereas *F-HF* would be the choice if it is believed to be greater than 0.75 (Barcikowski and Robey, 1984; Girden, 1992; Verma, 2016).

In summary, although these adjusted *F*-test procedures were developed several decades ago (between the 1950s and 1970s), controversy remains as to which procedure is best. This is illustrated by the fact that the recommendations found in classical methodology texts often differ from those derived from simulation studies. While most of the former, based on early studies, advise using *F-GG* over *F-HF* (as the former is more conservative), or state that the choice of strategy depends on the expected *ε* value, some more recent simulation studies suggest either that the two adjusted *F*-tests show equivalent performance or that *F-HF* outperforms *F-GG*. Consequently, although RM-ANOVA is the conventional analysis most commonly used with repeated measures, there are still no clear guidelines that can help applied researchers who wish to use this approach to choose the most appropriate adjusted *F*-test if sphericity is not satisfied.

It should also be noted that the aforementioned Monte Carlo studies have several limitations. First, they analyze a limited number of sphericity violation conditions, and hence the results may not be generalizable to other cases. For example, Berkovits et al. (2000) included only three conditions (0.48, 0.57, and 0.75) whereas Muller et al. (2007) analyzed two (0.28 and 0.51) and Oberfeld and Franke (2013) one (0.50). Second, some of the studies focused especially on small samples and with a high number of repeated measures, such as Muller et al. (2007) who analyzed 9 repeated measures with a maximum sample size of 40, and Haverkamp and Beauducel (2019) who included 9 and 12 repeated measures with sample sizes of 30 or less. In addition, most of them are aimed at comparing different procedures, such as the multivariate approach, the linear mixed model, bootstrapping and comparison of trimmed means, or structural equation models (e.g., Algina and Keselman, 1997; Berkovits et al., 2000; Wilcox et al., 2000; Muller et al., 2007; Voelkle and McKnight, 2012; Oberfeld and Franke, 2013; Langenberg et al., 2023). This makes it difficult for applied researchers to draw useful conclusions for their analyses.

The aim of the present study was therefore to extend knowledge from previous studies and to analyze extensively the effect of sphericity violation, irrespective of non-normality, on the *F*-statistic, *F-GG*, and *F-HF*, examining the performance of each in terms of Type I error and power under a variety of conditions that may be encountered in actual research practice. Normal data were generated with 3, 4, and 6 repeated measures, considering more sample size conditions (from 10 to 300, representing small, medium, and large samples) and a wider range of sphericity violation conditions than previous studies, including estimated values of *ε* ranging from its lower limit (most severe deviation from sphericity) to the upper limit (sphericity fulfilled). For the analysis of Type I error, we used Bradley’s (1978) liberal criterion as a generally appropriate strategy for conducting tests of mean difference in the absence of sphericity. Under this criterion, a procedure is robust if Type I error falls within the interval [2.5, 7.5]. To refine the results and obtain a more detailed understanding of the behavior of the statistics, we also interpreted robustness in terms of Bradley’s (1978) stringent criterion, which ensures that Type I error remains closer to 5%, in the interval [4.5, 5.5], for a nominal alpha of 5%. For power analysis, we added different mean patterns for each design, considering a medium effect size for each combination of estimations of *ε* and *K*. In total, we analyzed Type I error in 437 experimental conditions and power in 1,140 conditions. A key strength of the study is therefore that it yields results based on a large number of manipulated variables that represent more real-life situations than have been analyzed in previous research. Our overall goal was to use these results to clarify the performance of the *F*-statistic, *F-GG*, and *F-HF* and to establish practical recommendations for applied researchers who wish to use these procedures.

## Method

A Monte Carlo simulation study was performed using SAS 9.4 software with the IML (interactive matrix language) module. A series of macros was created that allowed generation of the data. Normal data were generated using the Cholesky transformation of the covariance matrix. This transformation simulates data that mimic the patterns generated by an unstructured covariance matrix. Matrices were generated with different estimated values of *ε*, which is defined by Equation (1) (Box, 1954; Geisser and Greenhouse, 1958; Greenhouse and Geisser, 1959) for a one-way repeated measures design (for an example, see Kirk, 2013, pp. 313–314):


(1) $$\hat{\varepsilon }=\frac{k^{2}{\left({\bar{s}}_{jj}-{\bar{s}}_{..}\right)}^{2}}{\left(k-1\right)\left(\sum_{j=1}^{k}\sum_{i=1}^{n}s_{jj´}^{2}-2k\sum_{j=1}^{k}{\bar{s}}_{j.}{\;}^{2}+k^{2}{\bar{s}}_{..}{\;}^{2}\right)}$$

where k = number of repeated measures, ${\bar{s}}_{jj}$= mean of elements on the main diagonal of the covariance matrix, ${\bar{s}}_{..}$= mean of all elements of the matrix, $\sum_{j=1}^{k}\sum_{i=1}^{n}s_{jj´}^{2}$ = sum of each element squared of the matrix, ${\bar{s}}_{j.}$= mean of elements in row *j*. We used this procedure to estimate the population value as it is more conservative than $\tilde{\varepsilon }$, and also because the calculation is available in most of the popular statistical packages (IBM-SPSS, SAS, R, etc.). Researchers may use the value obtained to decide upon the most suitable analytic strategy.

Simulated data were analyzed with PROC GLM of SAS to obtain the probability values associated with unadjusted *F*, *F-GG*, and *F-HF*. Ten thousand replications were performed for each condition.

### Type I error

We considered a one-way design and manipulated the following variables:

1. Within-subject levels (*K*). The repeated measures were *K* = 3, 4, and 6.
2. Total sample size. The sample sizes considered were 10, 15, 20, 25, 30, 40, 50, 60, 70, 80, 90, 100, 120, 150, 180, 210, 240, 270, and 300. Keselman et al. (1998) found that 55.3% of studies with repeated measures reported a sample size of 60 or fewer, although the range varied from 6 to 1,000. These sample sizes were also used by Blanca et al. (2023). Accordingly, we considered a wide range of sample sizes corresponding to small, medium, and large samples.
3. Sphericity. The values of $\hat{\varepsilon }$ ranged between the lower limit (which varies according to the number of repeated measurements) and 1. For *K* = 3, $\hat{\varepsilon }$ values were approximately 0.50, 0.60, 0.70, 0.80, 0.90, and 1; for *K* = 4, $\hat{\varepsilon }$ values were approximately 0.33, 0.40, 0.50, 0.60, 0.70, 0.80, 0.90, and 1; and for *K* = 6, $\hat{\varepsilon }$ values were approximately 0.20, 0.30, 0.40, 0.50, 0.60, 0.70, 0.80, 0.90, and 1. The exact values of epsilon are available as Supplementary material.

We computed empirical Type I error rates, represented by the percentage rejection of the null hypothesis when differences between repeated measures are set to zero at a significance level of 5%. Results were interpreted using Bradley’s (1978) liberal criterion, according to which a procedure is robust if the Type I error rate is between 2.5 and 7.5% for a nominal alpha of 5%. We also used Bradley’s (1978) stringent criterion in order to refine the results, considering a procedure as robust if the Type I error rate was between 4.5 and 5.5% for the same nominal alpha. If the Type I error rate is above the respective upper bound the procedure is considered liberal, whereas if it is below the respective lower bound it is considered conservative.

### Empirical power

To analyze empirical power, a specific effect represented by the means vector was added to the generated data to incorporate a desired effect size. Mean values were selected to give a medium effect size *f* equal to 0.25 in the simulated samples. We obtained sample values of effect size in the interval [0.23, 0.25] for each combination of *K*, $\hat{\varepsilon }$, and *N*. The manipulated variables were the same as for Type I error rates in terms of number of repeated measures, values of $\hat{\varepsilon }$, and sample size. In addition, we considered three mean patterns for each *K*. With *K* = 3, one of the means was different from the means of the other repeated measures (e.g., 1, 1, 2; 1, 2, 1). With *K* = 4 and 6, the means were manipulated so that (a) one was different from the rest (e.g., 1, 1, 1, 2; 1, 1, 1, 1, 1, 2), and (b) half were different and equal to each other (e.g., 1, 1, 2, 2; 1, 1, 1, 2, 2, 2). For all *K*, the means were also manipulated so that the increase between them was linear and proportional (e.g., 1, 1.75, 2.5, 3.25). We chose these patterns because they have been typically used in simulation studies (e.g., Kowalchuk and Keselman, 2001; Kowalchuk et al., 2004; Vallejo et al., 2004; Hayoz, 2007) and were considered to represent different real research situations.

We calculated empirical power rates based on the percentage rejection of the null hypothesis for each mean pattern at a significance level of 5%.

## Results

### Empirical type I error rates

**Figure 1:**
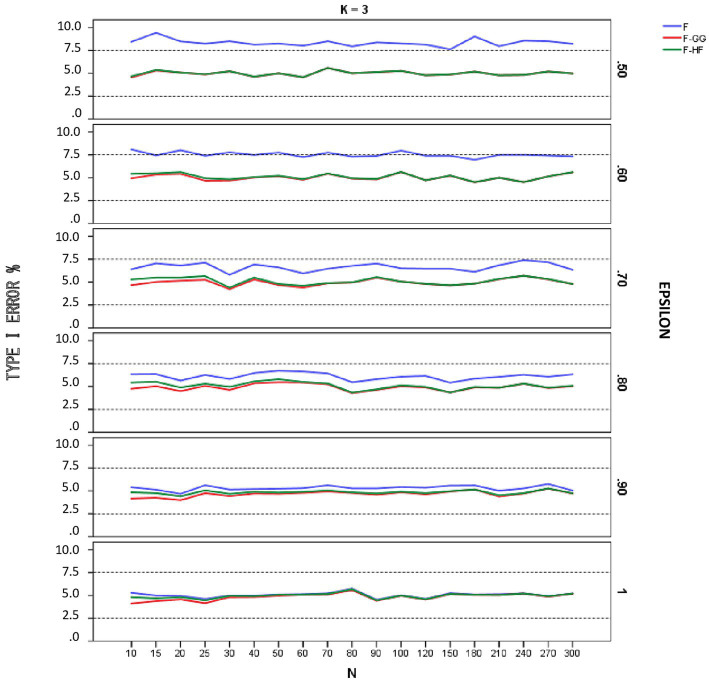
Type I error rates (in percentages) according to Bradley’s (1978) liberal criterion for the F-statistic and for F adjusted by the Greenhouse-Geisser (F-GG) and Huyhn-Feldt (F-HF) procedures as a function of epsilon (£) for *K* = 3.

**Figure 2:**
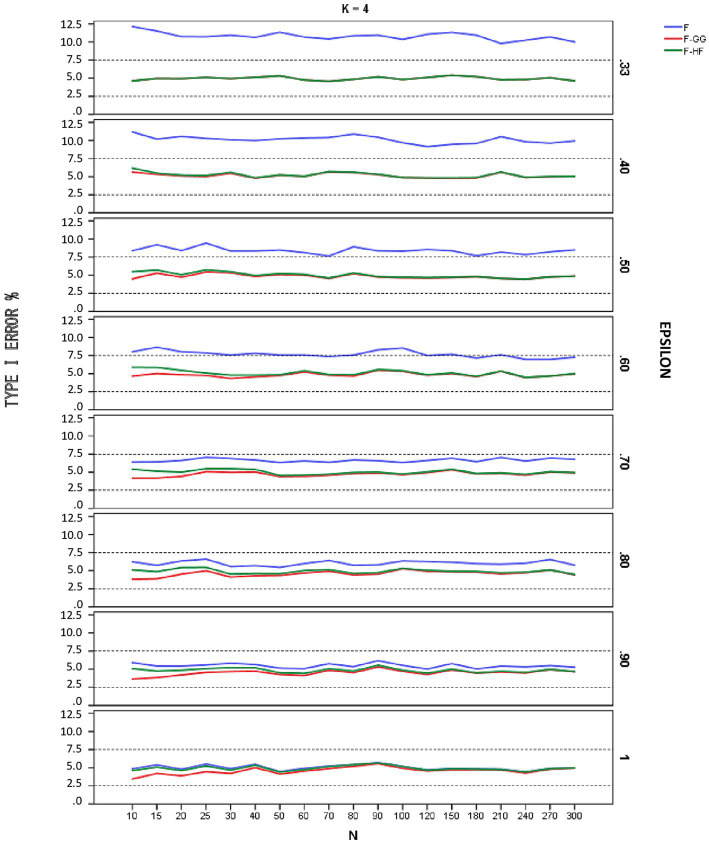
Type I error rates (in percentages) according to Bradley’s (1978) liberal criterion for the F-statistic and for F adjusted by the Greenhouse-Geisser (F-GG) and Huyhn-Feldt (F-HF) procedures as a function of epsilon (E) for *K* = 4.

**Figure 3:**
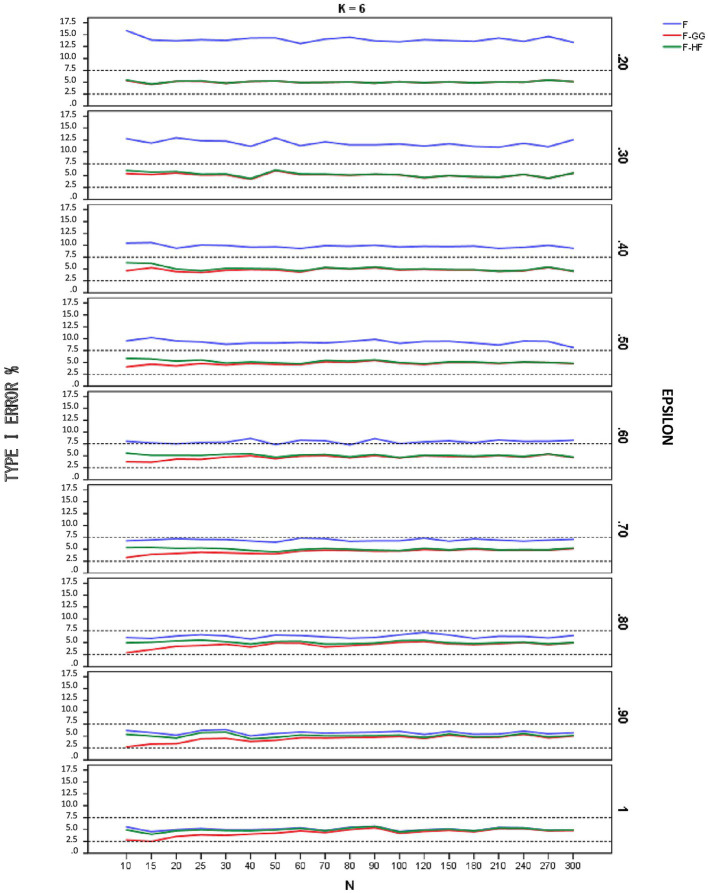
Type I error rates (in percentages) according to Bradley’s (1978) liberal criterion for the F-statistic and for F adjusted by the Greenhouse-Geisser (F-GG) and Huyhn-Feldt (F-HF) procedures as a function of epsilon (E) for *K* = 6.

Figures 1–3 show empirical Type I error rates according to Bradley’s (1978) liberal criterion for the *F*-statistic, *F-GG*, and *F-HF* as a function of sphericity for each *K*. Tables with the exact Type I error rates are available as Supplementary material. The results indicate that Type I error rates of *F-GG* and *F-HF* were always within the interval [2.5, 7.5] for considering a test as robust, although *F-GG* was more conservative, especially with large values of $\hat{\varepsilon }$ and small sample size. The findings also show that the *F*-statistic is liberal with values of $\hat{\varepsilon }$ below 0.70, regardless of sample size and *K*, and also that it becomes more liberal as the $\hat{\varepsilon }$ value decreases. With values of $\hat{\varepsilon }$ equal to or higher than 0.70, the *F*-statistic controls Type I error. However, although Type I error rates of the *F*-statistic were within the interval [2.5, 7.5], it shows a trend toward being more liberal than *F-GG* or *F-HF*, with rates around 6–7% up to a $\hat{\varepsilon }$ value of 0.90, whereas rates for the latter two tests are nearer 5%.

To further refine the results and be able to differentiate between the behavior of *F-GG* and *F-HF*, we also considered Bradley’s (1978) stringent criterion, which ensures that Type I error remains closer to 5%. Using this criterion, we computed the percentage robustness of the *F*-statistic, *F-GG*, and *F-HF* as a function of $\hat{\varepsilon }$ values across all sample sizes and values of *K*. The results are shown in Table 1. In this Table the results associated with the lower limit of $\hat{\varepsilon }$ for each *K* have been removed from the computation as they yield, as expected (Huynh and Feldt, 1976), the same estimated *ε* value and, therefore, the same results for *F-GG* and *F-HF*, reaching 100% robustness in all cases. The results show that *F-GG* tends to be more conservative, while *F-HF* tends to be more liberal. However, *F-GG* is more robust than *F-HF* for $\hat{\varepsilon }$ values below 0.60. By contrast, *F-HF* is superior to *F-GG* for $\hat{\varepsilon }$ values equal to or higher than 0.60. The F-statistic is only superior to the two adjusted tests when the $\hat{\varepsilon }$ value reaches 1.

**Table 1:** Percentage robustness of the *F*-statistic, *F-GG*, and *F-HF* according to Bradley’s stringent criterion.

		*F*			*F-GG*			*F-HF*	
$\hat{\varepsilon }$	Conservative	Robust	Liberal	Conservative	Robust	Liberal	Conservative	Robust	Liberal
0.30^a^	–	–	100	10.5	78.9	10.5	5.3	68.4	26.3
0.40^b^	–	–	100	5.3	84.2	10.5	–	81.6	18.4
0.50	–	–	100	7.9	92.1	–	–	84.2	15.8
0.60	–	–	100	12.3	84.2	3.5	–	87.7	12.3
0.70	–	–	100	24.6	73.7	1.8	1.8	93.0	5.3
0.80	–	5.3	94.7	28.1	71.9	–	3.5	89.5	7.0
0.90	–	54.4	45.6	29.8	70.2	–	7.0	86.0	7.0
1	1.8	93.0	5.3	35.1	61.4	3.5	5.3	89.5	5.3

Conservative: Type I error < 4.5; robust: falls in the interval [4.5, 5.5]; liberal: > 5.5. Shaded boxes indicate higher percentage robustness.

The results associated with the lower limit of $\hat{\varepsilon }$ for each K have been eliminated from the percentage computation because they yield the same estimated ε value and, therefore, the same results for F-GG and F-HF, reaching 100% robustness in all cases.

a For *K* = 6.

b For *K* = 4 and 6.

### Empirical power

**Figure 4:**
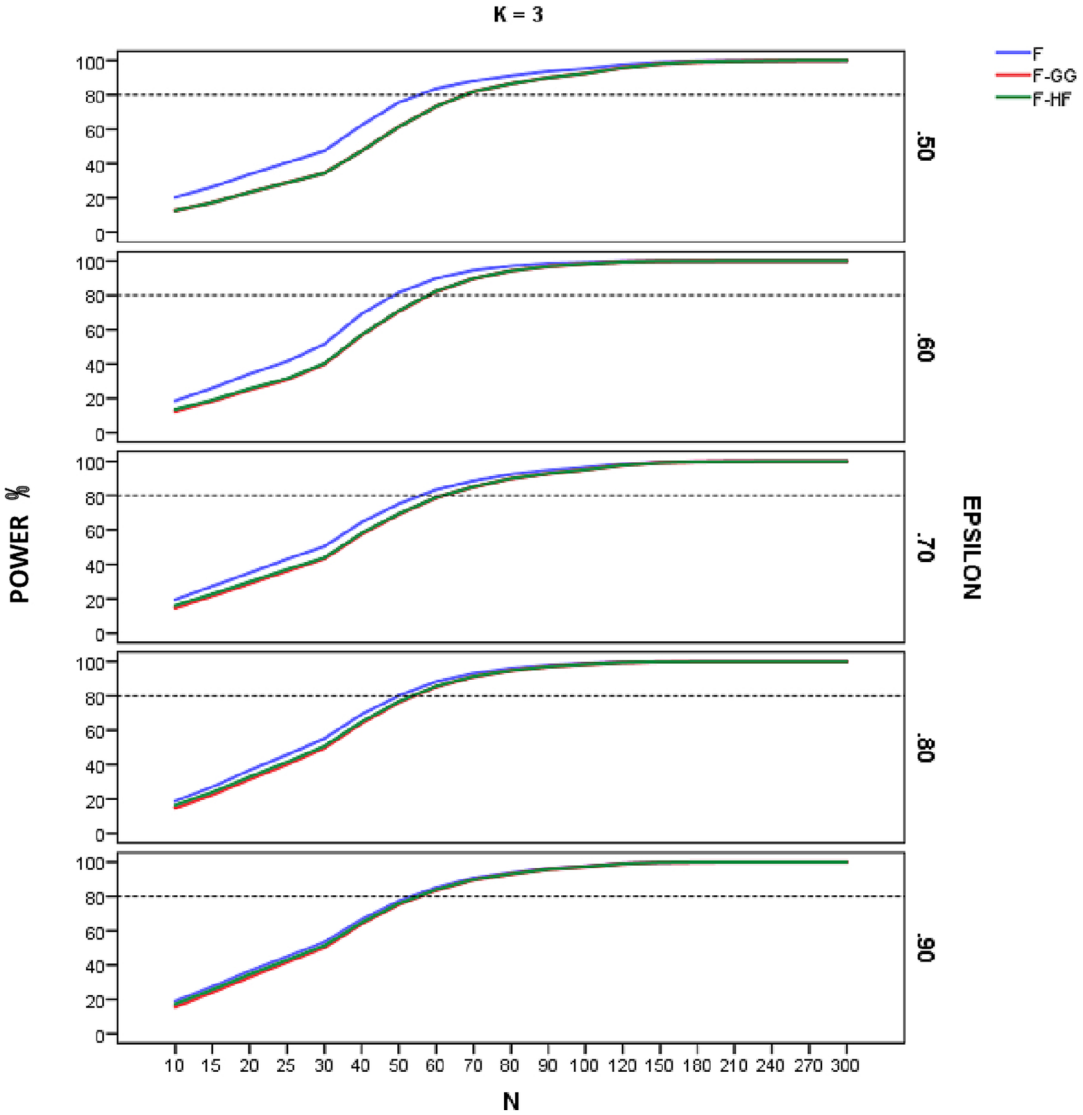
Empirical power of the F-statistic and of F adjusted by the Greenhouse-Geisser (F-GG) and Huyhn-Feldt (F-HF) procedures as a function of sphericity, sample size (N), and epsilon (£) for *K* = 3 across all mean pattern conditions.

**Figure 5:**
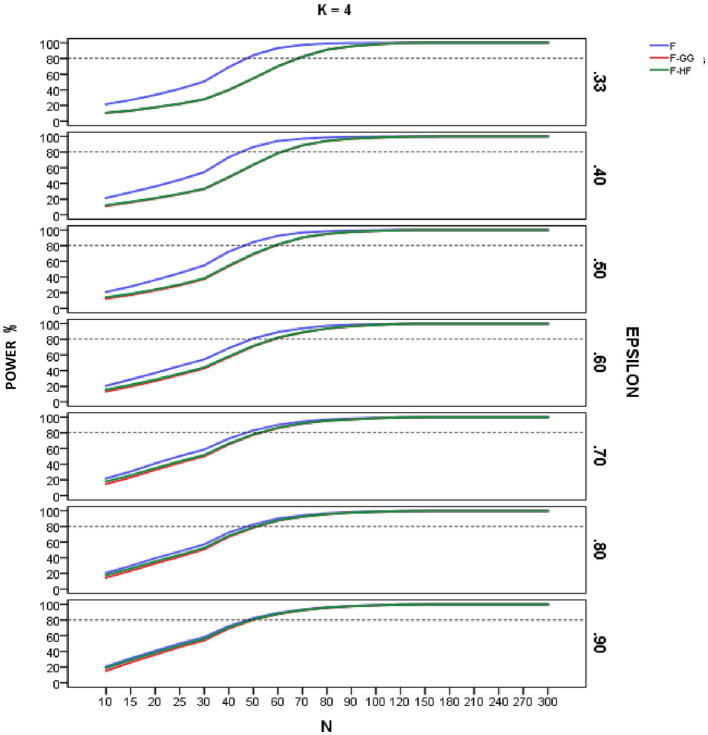
Empirical power of the F-statistic and of F adjusted by the Greenhouse-Geisser (F-GG) and Huyhn-Feldt (F-HF) procedures as a function of sphericity, sample size (N), and epsilon (E) for *K* = 4 across all mean pattern conditions.

**Figure 6:**
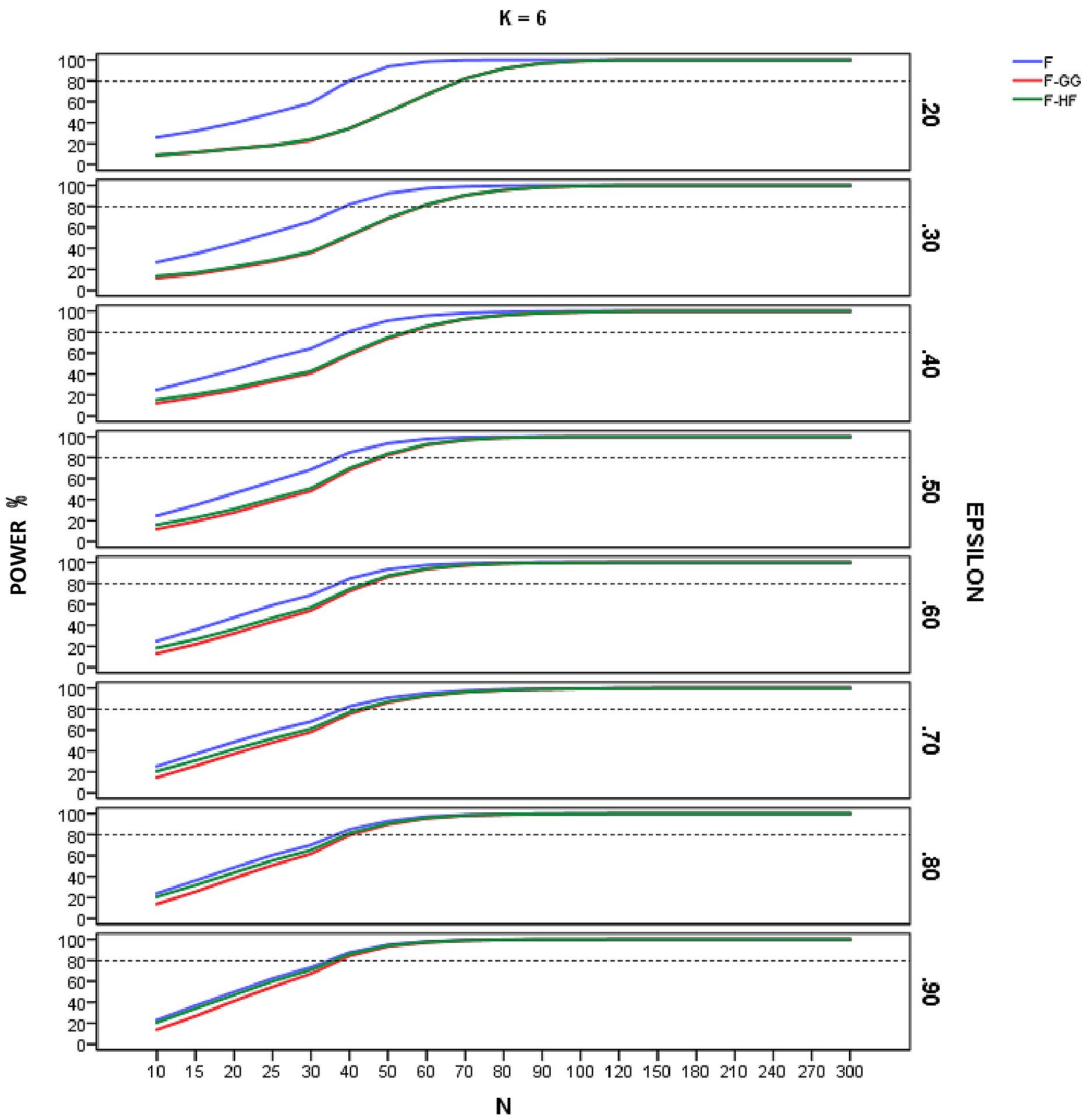
Empirical power of the F-statistic and of F adjusted by the Greenhouse-Geisser (F-GG) and Huyhn-Feldt (F-HF) procedures as a function of sphericity, sample size (N), and epsilon (E) for *K* = 6 across all mean pattern conditions.

Figures 4–6 show empirical power of the *F*-statistic, *F-GG*, and *F-HF* as a function of sample size and sphericity for each *K*. The mean patterns analyzed in this study did not significantly affect the empirical power, and hence the results are presented without taking this variable into account. Overall, power increases as sample size increases, the *F*-statistic is more powerful than the two adjusted tests, and *F-GG* and *F-HF* show similar power, although the former tends to show slightly lower power, especially with large values of $\hat{\varepsilon }$ and small sample size.

## Discussion

The aim of this study was to analyze the effect of sphericity violation, irrespective of non-normality, on the *F*-statistic, *F-GG*, and *F-HF*, examining the performance of each in terms of type I error and power under a variety of conditions that may be encountered in real research practice. Normal data were generated with 3, 4, and 6 repeated measures, considering different sample sizes with different degrees of sphericity violation according to the $\hat{\varepsilon }$ value, from most severe deviation to sphericity fulfilled. For power analysis, we added different mean patterns for each design, considering a medium effect size for each combination of $\hat{\varepsilon }$ and *K*. Our ultimate aim was to use the results to clarify the performance of these statistical procedures and to establish practical recommendations for applied researchers who wish to base their analysis on RM-ANOVA.

The results obtained when using Bradley’s (1978) liberal criterion show that in terms of Type I error the *F*-statistic is liberal with values of $\hat{\varepsilon }$ below 0.70, regardless of sample size and the number of repeated measures. The more severe the violation of sphericity, the more liberal the *F*-statistic is, even reaching a Type I error rate of 15.86% with an alpha of 5%. Therefore, this test should not be used with $\hat{\varepsilon }$ values below 0.70. With values of $\hat{\varepsilon }$ above 0.70, the *F*-statistic is robust, maintaining Type I error rates within the interval [2.5, 7.5], although these rates are systematically around 6–7%. With values of $\hat{\varepsilon }$ equal to 0.90, Type I error rates are closer to 5%, although some values are near 6%, especially for *K* = 6 and small sample sizes.

Regarding *F-GG* and *F-HF*, Type I error rates are always within the interval [2.5, 7.5] for considering a test as robust [according to Bradley’s (1978) liberal criterion], although the former is more conservative than the latter as $\hat{\varepsilon }$ increases, especially with small sample sizes. Overall, Type I error rates for the two adjusted *F*-tests are closer to 5% (the nominal alpha) than are those for the *F*-statistic in all conditions of sphericity violation. Therefore, both *F-HF* and *F-GG* may be adequate choices to correct the bias produced by violation of sphericity.

Overall, these results are in line with previous research that also used Bradley’s (1978) liberal criterion, showing the impact of sphericity violation on the robustness of the *F*-statistic (Box, 1954; Collier et al., 1967; Berkovits et al., 2000; Voelkle and McKnight, 2012; Haverkamp and Beauducel, 2017, 2019), and also that both adjusted *F*-tests provide reasonable control of Type I error under no sphericity (Berkovits et al., 2000; Muller et al., 2007). The tendency of *F-GG* to be more conservative has likewise been systematically found in previous research (Huynh and Feldt, 1976; Oberfeld and Franke, 2013; Haverkamp and Beauducel, 2017).

A more refined analysis of our results, using Bradley’s (1978) stringent criterion, allowed us to further investigate the behavior of the *F*-statistic, *F-GG*, and *F-HF*. This criterion ensures that Type I error remains closer to 5% than does the liberal criterion, specifically in the interval [4.5, 5.5]. The findings show that *F-GG* is more robust than *F-HF* for $\hat{\varepsilon }$ values below 0.60, whereas *F-HF* is superior to *F-GG* for $\hat{\varepsilon }$ values equal to or higher than 0.60. The *F*-statistic is only more robust that the two adjusted *F*-tests when $\hat{\varepsilon }$ is equal to 1. These findings suggest that with values of $\hat{\varepsilon }$ equal to or below 0.90, *F-GG* and *F-HF* outperform the *F*-statistic, leading us to propose a different strategy than that which follows from the results obtained with the liberal criterion. First, given that more robust alternative procedures are available, the *F*-statistic should not be used with an $\hat{\varepsilon }$ value of 0.90. Second, it would be advisable to use *F-GG* with $\hat{\varepsilon }$ values below 0.60, and *F-HF* when $\hat{\varepsilon }$ equal to or is above this value. This rule is very similar to the one suggested by Huynh and Feldt (1976) and recommended by other authors (Barcikowski and Robey, 1984; Girden, 1992; Verma, 2016), although the proposed cut-off point was higher (i.e., it was set at 0.75). Importantly, the present study has investigated more conditions of sphericity violation than was the case in previous research, and this has allowed a more accurate cut-off point to be established.

As for empirical power, this increased with increasing sample size across all the conditions studied, reflecting the well-known relationship between power and sample size. For sample sizes around 90–100, all three tests yield similar results, but with smaller samples the *F*-statistic is usually more powerful than are the adjusted *F*-tests. Its superiority is greater as the $\hat{\varepsilon }$ value decreases and, hence, as sphericity violation becomes severe. This result is logical and expected, given the higher Type I error rates of the *F*-statistic in these conditions. As the value of $\hat{\varepsilon }$ increases, the power superiority of the *F*-statistic becomes smaller. For example, in the case of $\hat{\varepsilon }$ equal to 0.90 there is almost no power advantage of the *F*-statistic over *F-HF* and *F-GG*. These results confirm the findings for Type I error and lead us to advise against using the *F*-statistic, even with $\hat{\varepsilon }$ of 0.90. The power analysis also showed that the empirical power of the two adjusted *F*-tests is similar, although *F-GG* has slightly lower power than *F-HF* as $\hat{\varepsilon }$ values increase. This may be explained by the fact that *F-GG* is more conservative than *F-HF* in these conditions.

The present study has a number of limitations that need to be acknowledged. First, the results may not be generalizable to factorial repeated measures designs, or to other scenarios where both normality and sphericity are violated. More extensive studies are warranted to clarify these issues. Second, although we introduced different mean patterns for each design in order to analyze power, only a medium effect size was considered. Future research should therefore include other conditions for this variable. Notwithstanding these limitations, this study makes an important contribution insofar as it considers a large number of manipulated conditions involving different numbers of repeated measures, different degrees of sphericity violation, and a large range of sample sizes. In addition, we consider both Type I error and power so to inform a strategy based on balance between the two. This approach enables us to propose practical recommendations for researchers that are applicable to a large number of conditions that may be encountered in real research.

### Practical recommendations

The results of this study allow us to propose several practical recommendations for researchers dealing with normal data and violation of sphericity in one-way RM-ANOVA. When using Bradley’s (1978) liberal criterion of robustness, which ensures that Type I error rates remain in the interval [2.5, 7.5] for a nominal alpha of 5%, both *F-GG* and *F-HF* control Type I error when sphericity is violated. Generally speaking, we recommend using *F-GG* because it is a more conservative test than is *F-HF*. However, this rule of thumb should be applied with caution, and if different statistical conclusions follow from application of the two tests with a given data set, we suggest using the one that is more reliable in terms of percentage robustness.

Applying a stricter criterion of robustness is especially useful when *F-GG* and *F-HF* yield discrepant results. Based on the results obtained when using Bradley’s (1978) stringent criterion, which ensures that Type I error remains closer to 5%, in the interval [4.5, 5.5], we propose using either *F-GG* or *F-HF*, depending on the $\hat{\varepsilon }$ value. In most statistical packages this estimation of *ε* is labeled as *Greenhouse–Geisser epsilon*, and if $\hat{\varepsilon }$ < 0.60, *F-GG* should be used, whereas if 0.60 ≤ $\hat{\varepsilon }$ ≤ 0.90, *F-HF* should be used.

Overall, if *F-GG* leads to rejection of the null hypothesis, we can reliably conclude that the treatment effect is statistically significant, as *F-HF* will give the same result. However, if *F-GG* suggests that the null hypothesis should be accepted, but *F-HF* rejects it, then we should trust in *F-GG* if $\hat{\varepsilon }$ < 0.60 and in *F-HF* if 0.60 ≤ $\hat{\varepsilon }$ ≤ 0.90. The advantage of this guideline is that it may eliminate the need to conduct Mauchly’s test as a preliminary analysis, basing the choice of analytic strategy on the value of $\hat{\varepsilon }$ instead.

Given that more robust alternative procedures are available, the *F*-statistic should not be used when sphericity is violated, even with a $\hat{\varepsilon }$ value equal to 0.90. With $\hat{\varepsilon }$ values above 0.90, the robustness of the *F*-statistic is likely to be higher and similar to the robustness of *F-GG*. Whatever the case, the present results indicate that if $\hat{\varepsilon }\infty$ is equal to 1, the *F*-statistic should be used as it is the most robust.

Finally, regarding power, the results show that as the value of $\hat{\varepsilon }$ decreases, a large sample size is necessary for both *F-GG* and *F-HF* to produce an empirical power of 0.80 for a medium effect size, which is the conventional threshold suggested when sample size is estimated *a priori* (Kirk, 2013; Cooper and Garson, 2016). This *a priori* power analysis is recommended in order to optimize the resources to be used in research (Faul et al., 2007). The increasing of sample size, if possible, may also be a strategy to compensate for anticipated violations of sphericity.

## Data availability statement

The original contributions presented in the study are included in the article/Supplementary material, further inquiries can be directed to the corresponding author.

## Author contributions

MB and RB contributed to conception and design of the study. JA and RB developed the SAS macros. MB, RA, and FG-C supervised the simulations, organized the database, and wrote the first draft of the manuscript. RB wrote sections of the manuscript. MB, JA, FG-C, RA, and RB participated in the final writing of the manuscript. All authors contributed to the article and approved the submitted version.

## Funding

This research was supported by University of Malaga and grant PID2020-113191GB-I00 from the MCIN/AEI/10.13039/501100011033.

## Conflict of interest

The authors declare that the research was conducted in the absence of any commercial or financial relationships that could be construed as a potential conflict of interest.

## Publisher’s note

All claims expressed in this article are solely those of the authors and do not necessarily represent those of their affiliated organizations, or those of the publisher, the editors and the reviewers. Any product that may be evaluated in this article, or claim that may be made by its manufacturer, is not guaranteed or endorsed by the publisher.

## References

[ref1] Algina J. Keselman H. (1997). Detecting repeated measures effects with univariate and multivariate statistics. Psychol. Methods 2, 208–218. doi: 10.1037/1082-989X.2.2.208

[ref2] Barcikowski R. S. Robey R. R. (1984). Decisions in single group repeated measures analysis: statistical tests and three computer packages. Am. Stat. 38, 148–150.

[ref3] Berkovits I. Hancock G. Nevitt J. (2000). Bootstrap resampling approaches for repeated measure designs: relative robustness to sphericity and normality violations. Educ. Psychol. Meas. 60, 877–892. doi: 10.1177/00131640021970961

[ref4] Blanca M. Alarcón R. Bono R. (2018). Current practices in data analysis procedures in psychology: what has changed? Front. Psychol. 9:2558. doi: 10.3389/fpsyg.2018.02558, PMID: 30618979 PMC6300498

[ref5] Blanca M. J. Arnau J. García-Castro F. J. Alarcón R. Bono R. (2023). Non-normal data in repeated measures: impact on type I error and power. Psicothema 35, 21–29. doi: 10.7334/psicothema2022.292, PMID: 36695847

[ref6] Box G. E. P. (1954). Some theorems on quadratic forms applied in the study of analysis of variance problems II. Effect of inequality of variance and of correlation of error in the two-way classification. Ann. Math. Stat. 25, 484–498. doi: 10.1214/aoms/1177728717

[ref7] Bradley J. V. (1978). Robustness? Br. J. Math. Stat. Psychol. 31, 144–152. doi: 10.1111/j.2044-8317.1978.tb00581.x

[ref8] Collier R. O. Baker F. B. Mandeville G. K. Hayes T. F. (1967). Estimates of test size for several test procedures based on conventional variance ratios in the repeated measures design. Psychometrika 32, 339–353. doi: 10.1007/BF02289596, PMID: 5234710

[ref9] Cooper J. A. Garson G. D. (2016). Power analysis. Asheboro, NC: Statistical Associates Blue Book Series.

[ref10] Counsell A. Harlow L. L. (2017). Reporting practices and use of quantitative methods in Canadian journal articles in psychology. Can. Psychol. 58, 140–147. doi: 10.1037/cap0000074, PMID: 28684887 PMC5494980

[ref11] Faul F. Erdfelder E. Lang A. G. Buchner A. (2007). G*power 3: a flexible statistical power analysis program for the social, behavioral, and biomedical sciences. Behav. Res. Methods 39, 175–191. doi: 10.3758/bf03193146, PMID: 17695343

[ref12] Fernández P. Vallejo G. Livacic-Rojas P. Herrero J. Cuesta M. (2010). Comparative robustness of six tests in repeated measures designs with specified departures from sphericity. Qual. Quant. 44, 289–301. doi: 10.1007/s11135-008-9198-3

[ref13] Gamst G. Meyers L. Guarino A. J. (2008). Analysis of variance designs: A conceptual and computational approach with SPSS and SAS. New York, NY: Cambridge University Press.

[ref14] Geisser S. W. Greenhouse S. (1958). An extension of Box’s results on the use of the F distribution in multivariate analysis. Ann. Math. Stat. 29, 885–891. doi: 10.1214/aoms/1177706545

[ref15] Girden E. R. (1992). ANOVA repeated measures. Newbury Park, CA: Sage University Press.

[ref16] Goedert K. Boston R. Barrett A. (2013). Advancing the science of spatial neglect rehabilitation: an improved statistical approach with mixed linear modeling. Front. Hum. Neurosci. 7:211. doi: 10.3389/fnhum.2013.00211, PMID: 23730283 PMC3657689

[ref17] Greenhouse S. W. Geisser S. (1959). On methods in the analysis of profile data. Psychometrika 24, 95–112. doi: 10.1007/BF02289823

[ref18] Haverkamp N. Beauducel A. (2017). Violation of the sphericity assumption and its effect on type-I error rates in repeated measures ANOVA and multi-level linear models (MLM). Front. Psychol. 8:1841. doi: 10.3389/fpsyg.2017.01841, PMID: 29089917 PMC5651023

[ref19] Haverkamp N. Beauducel A. (2019). Differences of type I error rates for ANOVA and multilevel-linear-models using SAS and SPSS for repeated measures designs. Meta Psychol. 3:MP.2018.898. doi: 10.15626/mp.2018.898

[ref20] Hayoz S. (2007). Behavior of nonparametric tests in longitudinal design. 15th European young statisticians meeting Available at: http://matematicas.unex.es/~idelpuerto/WEB_EYSM/Articles/ch_stefanie_hayoz_art.pdf

[ref21] Huynh H. Feldt L. S. (1976). Estimation of the Box correction for degrees of freedom from sample data in randomized block and split-plot designs. J. Educ. Stat. 1, 69–82. doi: 10.2307/1164736

[ref22] Huynh H. Mandeville G. K. (1979). Validity conditions in repeated measures designs. Psychol. Bull. 86, 964–973. doi: 10.1037/0033-2909.86.5.964

[ref23] Kashy D. A. Donnellan M. B. Ackerman R. A. Russell D. W. (2009). Reporting and interpreting research in PSPB: practices, principles, and pragmatics. Personal. Soc. Psychol. Bull. 35, 1131–1142. doi: 10.1177/0146167208331253, PMID: 19458094

[ref24] Keselman H. J. Algina J. Kowalchuk R. K. Wolfinger R. D. (1999). A comparison of recent approaches to the analysis of repeated measurements. Br. J. Math. Stat. Psychol. 52, 63–78. doi: 10.1348/000711099158964

[ref25] Keselman H. J. Huberty C. Lix L. Olejnik S. Cribbie R. Donahue B.. (1998). Statistical practices of educational researchers: an analysis of their ANOVA, MANOVA, and ANCOVA analyses. Rev. Educ. Res. 68, 350–386. doi: 10.3102/00346543068003350

[ref26] Keselman J. C. Keselman H. J. (1990). Analysing unbalanced repeated measures designs. Br. J. Math. Stat. Psychol. 43, 265–282. doi: 10.1111/j.2044-8317.1990.tb00940.x

[ref27] Keselman H. J. Keselman J. C. Lix L. M. (1995). The analysis of repeated measures: univariate test, multivariate, or both? Br. J. Math. Stat. Psychol. 48, 319–338. doi: 10.1111/j.2044-8317.1995.tb01066.x

[ref28] Keselman H. J. Rogan J. C. Mendoza J. L. Breen L. J. (1980). Testing the validity conditions of repeated measures F tests. Psychol. Bull. 87, 479–481. doi: 10.1037/0033-2909.87.3.479

[ref29] Kirk R. E. (2013). Experimental design. Procedures for the behavioral sciences. 4th Edn. Thousand Oaks, CA: Sage Publications.

[ref30] Kowalchuk R. K. Keselman H. J. (2001). Mixed-model pairwise multiple comparisons of repeated measures means. Psychol. Bull. 6, 282–296. doi: 10.1037/1082-989x.6.3.282, PMID: 11570233

[ref31] Kowalchuk R. K. Keselman H. J. Algina J. Wolfinger R. D. (2004). The analysis of repeated measurements with mixed-model adjusted F tests. Educ. Psychol. Meas. 64, 224–242. doi: 10.1177/0013164403260196

[ref32] Langenberg B. Helm J. L. Günther T. Mayer A. (2023). Understanding, testing, and relaxing sphericity of repeated measures ANOVA with manifest and latent variables using SEM. Methodology 19, 60–95. doi: 10.5964/meth.8415

[ref33] Maxwell S. E. Delaney H. D. (2004). Designing experiments and analyzing data: A model comparison perspective. 2nd Edn. Belmont, CA: Lawrence Erlbaum Associates.

[ref34] Muller K. Edwards L. Simpson S. Taylor D. (2007). Statistical tests with accurate size and power for balanced linear mixed models. Stat. Med. 26, 3639–3660. doi: 10.1002/sim.2827, PMID: 17394132

[ref35] Oberfeld D. Franke T. (2013). Evaluating the robustness of repeated measures analyses: the case of small sample sizes and nonnormal data. Behav. Res. Methods 45, 792–812. doi: 10.3758/s13428-012-0281-2, PMID: 23184532

[ref36] Quintana S. M. Maxwell S. E. (1994). A Monte Carlo comparison of seven ε-adjustment procedures in repeated measures designs with small sample sizes. J. Educ. Stat. 19, 57–71. doi: 10.3102/10769986019001057

[ref37] Tabachnick B. G. Fidell L. (2007). Experimental designs using ANOVA. Belmont, CA: Thomson.

[ref38] Vallejo G. Fernández P. Herrero F. J. Conejo N. M. (2004). Alternative procedures for testing fixed effects in repeated measures designs when assumptions are violated. Psicothema 16, 498–508.

[ref39] Verma J. P. (2016). Repeated measures design for empirical researchers. New Jersey: Wiley.

[ref40] Voelkle M. C. McKnight P. E. (2012). One size fits all? A Monte-Carlo simulation on the relationship between repeated measures (M)ANOVA and latent curve modeling. Methodol. Eur. J. Res. Methods Behav. Soc. Sci. 8, 23–38. doi: 10.1027/1614-2241/a000044

[ref41] Wilcox R. R. Keselman H. J. Muska J. Cribbie R. (2000). Repeated measures ANOVA: some new results on comparing trimmed means and means. Br. J. Math. Stat. Psychol. 53, 69–82. doi: 10.1348/000711000159187, PMID: 10895523

