# Supplementary material for: Repeated measures ANOVA and adjusted F-tests when sphericity is violated: which procedure is best?
Source: Front Psychol. 2023 Aug 30;14:1192453. doi: 10.3389/fpsyg.2023.1192453 (PMC10499170; doi:10.3389/fpsyg.2023.1192453)
Supplement: Supplementary file 1 [file Data_Sheet_1.PDF]

Repeated Measures ANOVA and Adjusted *F*-Tests When Sphericity is Violated: Which Procedure is Best?

Blanca, M.J., Arnau, J., García-Castro, J. F., Alarcón, J., & Bono, R.

Exact values of ε:

K = 3: ε = .50516525, .60098722, .70276994, .80099907, .90012654, 1

K = 4; ε = .34791979, .40126561, .50052295, .60785892, .70991146, .80098645, .90084738, 1

K = 6; ε = .2081422, .30134424, .4008269, .5027216, .5985405, .7090667, .8007163, .9036866, 1

Table 1. Type I error rates (in percentages) for the *F*-statistic and for *F* adjusted by the Greenhouse-Geisser (*F-GG*) and Huyhn-Feldt (*F-HF*) procedures as a function of epsilon (ε) for $K = 3$. Type I error rates outside the boundary of Bradley's liberal criterion are in bold (conservative: < 2.5; liberal: > 7.5).

	$\varepsilon = .50$			$\varepsilon = .60$			$\varepsilon = .70$			$\varepsilon = .80$			$\varepsilon = .90$			$\varepsilon = 1$		
N	*F*	*F-GG*	*F-HF*	*F*	*F-GG*	*F-HF*	*F*	*F-GG*	*F-HF*	*F*	*F-GG*	*F-HF*	*F*	*F-GG*	*F-HF*	*F*	*F-GG*	*F-HF*
10	**8.44**	4.56	4.68	**8.08**	4.94	5.42	6.40	4.68	5.30	6.34	4.76	5.44	5.40	4.18	4.84	5.28	4.12	4.80
15	**9.42**	5.30	5.36	7.44	5.34	5.48	7.06	5.02	5.50	6.36	5.04	5.52	5.14	4.26	4.76	4.98	4.38	4.68
20	**8.48**	5.08	5.08	**8.00**	5.42	5.62	6.82	5.16	5.50	5.64	4.50	4.90	4.68	4.00	4.42	4.94	4.56	4.80
25	**8.24**	4.88	4.90	7.40	4.68	4.94	7.14	5.26	5.66	6.28	5.08	5.30	5.62	4.76	5.06	4.60	4.16	4.46
30	**8.50**	5.22	5.22	**7.76**	4.70	4.82	5.80	4.24	4.40	5.82	4.64	4.96	5.16	4.44	4.68	4.98	4.80	4.94
40	**8.14**	4.62	4.64	7.48	5.02	5.06	6.94	5.30	5.50	6.48	5.36	5.56	5.20	4.72	4.90	4.96	4.82	4.92
50	**8.24**	5.00	5.00	**7.74**	5.16	5.22	6.60	4.68	4.80	6.72	5.46	5.78	5.24	4.70	4.82	5.08	4.96	5.06
60	**8.02**	4.56	4.58	7.26	4.78	4.84	5.96	4.40	4.60	6.64	5.44	5.50	5.30	4.80	4.90	5.12	5.06	5.08
70	**8.50**	5.58	5.58	**7.74**	5.44	5.46	6.46	4.88	4.90	6.44	5.26	5.34	5.62	4.94	5.06	5.22	5.08	5.16
80	**7.94**	5.00	5.00	7.32	4.90	4.94	6.78	4.96	4.98	5.46	4.30	4.36	5.28	4.78	4.84	5.72	5.58	5.68
90	**8.38**	5.12	5.14	7.38	4.82	4.86	7.04	5.48	5.56	5.80	4.64	4.70	5.28	4.60	4.72	4.52	4.42	4.46
100	**8.26**	5.26	5.26	**7.96**	5.62	5.64	6.50	5.06	5.06	6.08	5.02	5.14	5.44	4.86	4.92	5.00	4.96	4.98
120	**8.14**	4.78	4.78	7.40	4.72	4.72	6.46	4.80	4.82	6.16	4.90	4.96	5.36	4.64	4.76	4.60	4.54	4.58
150	**7.62**	4.86	4.88	7.40	5.22	5.24	6.46	4.64	4.66	5.42	4.36	4.36	5.58	4.94	4.98	5.24	5.14	5.16
180	**9.02**	5.18	5.18	6.96	4.50	4.52	6.12	4.84	4.84	5.86	4.90	4.96	5.60	5.16	5.16	5.08	5.04	5.06
210	**7.96**	4.78	4.78	7.50	5.00	5.00	6.86	5.32	5.36	6.06	4.88	4.88	5.02	4.40	4.52	5.12	5.04	5.04
240	**8.56**	4.82	4.82	7.50	4.52	4.52	7.40	5.70	5.70	6.30	5.30	5.32	5.28	4.72	4.76	5.20	5.20	5.20
270	**8.50**	5.20	5.20	7.42	5.14	5.14	7.18	5.30	5.34	6.08	4.84	4.88	5.76	5.26	5.28	4.90	4.86	4.90
300	**8.22**	4.98	4.98	7.34	5.60	5.62	6.34	4.80	4.80	6.34	5.04	5.08	5.04	4.72	4.74	5.20	5.18	5.20

Table 2. Type I error rates (in percentages) for the *F*-statistic and for *F* adjusted by the Greenhouse-Geisser (*F-GG*) and Huyhn-Feldt (*F-HF*) procedures as a function of epsilon (ε) for $K = 4$. Type I error rates outside the boundary of Bradley's liberal criterion are in bold (conservative: < 2.5; liberal: > 7.5).

	$\varepsilon = .33$			$\varepsilon = .40$			$\varepsilon = .50$			$\varepsilon = .60$			$\varepsilon = .70$			$\varepsilon = .80$			$\varepsilon = .90$			$\varepsilon = 1$		
N	*F*	*F-GG*	*F-HF*	*F*	*F-GG*	*F-HF*	*F*	*F-GG*	*F-HF*	*F*	*F-GG*	*F-HF*	*F*	*F-GG*	*F-HF*	*F*	*F-GG*	*F-HF*	*F*	*F-GG*	*F-HF*	*F*	*F-GG*	*F-HF*
10	**12.14**	4.62	4.62	**11.20**	5.62	6.18	**8.38**	4.48	5.48	**8.02**	4.66	5.88	6.40	4.18	5.40	6.22	3.82	5.10	5.90	3.64	5.08	4.86	3.44	4.62
15	**11.50**	4.96	4.96	**10.18**	5.32	5.48	**9.20**	5.28	5.70	**8.64**	5.02	5.86	6.44	4.18	5.12	5.74	3.88	4.86	5.42	3.84	4.74	5.38	4.20	5.08
20	**10.74**	4.94	4.94	**10.56**	5.12	5.20	**8.40**	4.72	5.06	**8.02**	4.84	5.46	6.62	4.42	5.00	6.34	4.52	5.44	5.42	4.22	4.86	4.80	3.88	4.62
25	**10.72**	5.10	5.10	**10.28**	5.04	5.16	**9.42**	5.44	5.72	**7.88**	4.76	5.08	7.04	5.06	5.48	6.58	4.98	5.46	5.58	4.56	5.08	5.48	4.44	5.24
30	**10.94**	4.94	4.94	**10.10**	5.46	5.60	**8.34**	5.34	5.48	**7.58**	4.32	4.80	6.88	4.98	5.48	5.58	4.12	4.52	5.82	4.66	5.20	4.88	4.20	4.68
40	**10.64**	5.12	5.12	**10.00**	4.80	4.84	**8.34**	4.80	4.94	**7.82**	4.56	4.80	6.68	5.02	5.36	5.70	4.32	4.60	5.64	4.72	5.18	5.44	5.02	5.34
50	**11.34**	5.32	5.32	**10.22**	5.20	5.24	**8.48**	5.10	5.22	**7.58**	4.74	4.84	6.34	4.40	4.56	5.46	4.36	4.58	5.14	4.28	4.50	4.46	4.12	4.40
60	**10.68**	4.74	4.74	**10.34**	5.04	5.06	**8.12**	5.00	5.12	**7.58**	5.24	5.42	6.56	4.44	4.60	5.98	4.68	5.00	5.08	4.14	4.42	4.92	4.52	4.74
70	**10.42**	4.56	4.56	**10.40**	5.66	5.70	**7.64**	4.54	4.60	7.36	4.78	4.88	6.38	4.58	4.72	6.40	4.92	5.12	5.76	4.84	5.06	5.22	4.88	5.16
80	**10.86**	4.84	4.84	**10.90**	5.56	5.62	**8.92**	5.22	5.30	**7.58**	4.66	4.84	6.68	4.80	4.96	5.74	4.42	4.62	5.34	4.52	4.74	5.40	5.20	5.40
90	**10.94**	5.18	5.18	**10.44**	5.28	5.32	**8.36**	4.74	4.80	**8.28**	5.50	5.58	6.58	4.90	5.00	5.80	4.52	4.72	6.18	5.32	5.54	5.70	5.56	5.66
100	**10.36**	4.82	4.82	**9.68**	4.88	4.88	**8.32**	4.64	4.70	**8.54**	5.32	5.42	6.34	4.64	4.74	6.34	5.26	5.32	5.52	4.70	4.86	5.16	4.92	5.16
120	**11.06**	5.08	5.08	**9.16**	4.82	4.84	**8.54**	4.60	4.66	**7.52**	4.78	4.84	6.62	4.94	5.04	6.26	4.92	5.04	5.02	4.28	4.44	4.68	4.54	4.66
150	**11.32**	5.42	5.42	**9.46**	4.78	4.84	**8.38**	4.66	4.72	**7.68**	5.02	5.10	6.92	5.32	5.40	6.16	4.82	4.92	5.76	4.90	5.00	4.88	4.72	4.84
180	**10.94**	5.20	5.20	**9.58**	4.82	4.88	**7.68**	4.78	4.80	7.16	4.58	4.64	6.46	4.76	4.82	5.96	4.78	4.90	5.04	4.46	4.50	4.80	4.74	4.80
210	**9.80**	4.78	4.78	**10.52**	5.60	5.64	**8.18**	4.54	4.56	**7.60**	5.34	5.36	7.02	4.82	4.92	5.90	4.56	4.70	5.42	4.62	4.72	4.78	4.68	4.76
240	**10.24**	4.82	4.82	**9.82**	4.88	4.90	**7.84**	4.44	4.46	6.98	4.44	4.48	6.54	4.58	4.70	6.02	4.70	4.78	5.32	4.48	4.56	4.44	4.26	4.40
270	**10.70**	5.06	5.06	**9.62**	4.98	5.02	**8.22**	4.76	4.76	6.98	4.68	4.68	6.94	5.02	5.06	6.54	5.08	5.10	5.48	4.94	4.98	4.88	4.80	4.88
300	**10.00**	4.62	4.62	**9.94**	5.04	5.04	**8.50**	4.88	4.88	7.26	4.94	5.02	6.78	4.92	4.94	5.76	4.42	4.46	5.28	4.64	4.66	4.98	4.96	4.96

Table 3. Type I error rates (in percentages) for the *F*-statistic and for *F* adjusted by the Greenhouse-Geisser (*F-GG*) and Huyhn-Feldt (*F-HF*) procedures as a function of epsilon (ε) for $K = 6$. Type I error rates outside the boundary of Bradley's liberal criterion are in bold (conservative: < 2.5; liberal: > 7.5).

	$\varepsilon = .20$			$\varepsilon = .30$			$\varepsilon = .40$			$\varepsilon = .50$			$\varepsilon = .60$		
N	*F*	*F-GG*	*F-HF*	*F*	*F-GG*	*F-HF*	*F*	*F-GG*	*F-HF*	*F*	*F-GG*	*F-HF*	*F*	*F-GG*	*F-HF*
10	**15.86**	5.32	5.46	**12.76**	5.42	6.06	**10.46**	4.66	6.34	**9.54**	4.08	5.86	**8.08**	3.76	5.56
15	**13.88**	4.54	4.60	**11.84**	5.22	5.74	**10.56**	5.28	6.20	**10.22**	4.60	5.72	**7.74**	3.68	5.14
20	**13.70**	5.20	5.20	**12.96**	5.56	5.86	**9.38**	4.48	5.00	**9.50**	4.28	5.28	**7.54**	4.32	5.14
25	**13.92**	5.18	5.28	**12.34**	5.10	5.30	**10.06**	4.28	4.66	**9.32**	4.78	5.52	**7.82**	4.28	5.12
30	**13.80**	4.78	4.80	**12.26**	5.16	5.34	**9.96**	4.74	5.14	**8.84**	4.48	4.86	**7.88**	4.74	5.36
40	**14.30**	5.16	5.18	**11.14**	4.24	4.38	**9.60**	4.88	5.10	**9.08**	4.78	5.10	**8.66**	5.04	5.44
50	**14.32**	5.24	5.26	**12.92**	6.02	6.18	**9.66**	4.82	5.04	**9.08**	4.58	4.90	7.36	4.40	4.70
60	**13.14**	4.92	4.92	**11.28**	5.20	5.34	**9.32**	4.40	4.56	**9.22**	4.54	4.74	**8.30**	4.98	5.20
70	**14.06**	4.96	4.96	**12.10**	5.20	5.32	**9.92**	5.24	5.32	**9.10**	5.16	5.42	**8.18**	5.06	5.32
80	**14.46**	5.06	5.06	**11.46**	5.06	5.12	**9.80**	5.00	5.06	**9.42**	5.04	5.24	7.32	4.58	4.80
90	**13.68**	4.82	4.82	**11.46**	5.24	5.30	**10.00**	5.30	5.44	**9.84**	5.42	5.56	**8.62**	5.08	5.32
100	**13.50**	5.10	5.10	**11.66**	5.14	5.18	**9.64**	4.80	4.92	**9.02**	4.82	4.96	**7.60**	4.56	4.62
120	**13.92**	4.88	4.90	**11.20**	4.54	4.58	**9.78**	4.92	5.04	**9.42**	4.56	4.74	**7.96**	5.04	5.14
150	**13.74**	5.08	5.08	**11.70**	5.00	5.00	**9.74**	4.82	4.90	**9.44**	4.98	5.12	**8.16**	4.90	5.06
180	**13.58**	4.86	4.86	**11.12**	4.70	4.76	**9.82**	4.82	4.88	**9.08**	4.98	5.10	**7.78**	4.80	4.92
210	**14.30**	5.06	5.06	**10.98**	4.60	4.68	**9.34**	4.54	4.54	**8.70**	4.76	4.84	**8.36**	5.08	5.16
240	**13.56**	5.02	5.02	**11.80**	5.22	5.24	**9.56**	4.62	4.72	**9.48**	5.04	5.08	**8.06**	4.74	4.88
270	**14.64**	5.44	5.44	**11.06**	4.44	4.46	**9.98**	5.34	5.42	**9.42**	4.96	4.98	**8.08**	5.38	5.40
300	**13.38**	5.12	5.12	**12.56**	5.50	5.52	**9.38**	4.54	4.58	**8.14**	4.74	4.82	**8.30**	4.64	4.74

Table 3 (continued). Type I error rates (in percentages) for the *F*-statistic and for *F* adjusted by the Greenhouse-Geisser (*F-GG*) and Huyhn-Feldt (*F-HF*) procedures as a function of epsilon (ε) for $K = 6$. Type I error rates outside the boundary of Bradley's liberal criterion are in bold (conservative: < 2.5; liberal: > 7.5).

	$\varepsilon = .70$			$\varepsilon = .80$			$\varepsilon = .90$			$\varepsilon = 1$		
N	*F*	*F-GG*	*F-HF*	*F*	*F-GG*	*F-HF*	*F*	*F-GG*	*F-HF*	*F*	*F-GG*	*F-HF*
10	6.78	3.28	5.34	6.04	2.88	4.96	6.16	2.72	5.36	5.50	2.80	4.90
15	6.98	3.90	5.38	5.90	3.54	5.08	5.74	3.32	5.00	4.54	2.52	4.02
20	7.24	4.12	5.22	6.40	4.24	5.34	5.18	3.38	4.60	4.96	3.54	4.74
25	7.10	4.36	5.28	6.68	4.42	5.54	6.18	4.44	5.74	5.18	3.88	4.94
30	7.08	4.24	5.12	6.44	4.68	5.18	6.34	4.52	5.82	4.94	3.78	4.78
40	6.76	4.12	4.72	5.76	4.10	4.70	5.02	3.88	4.44	4.94	4.04	4.74
50	6.48	4.04	4.46	6.62	4.94	5.24	5.56	4.12	4.72	5.04	4.26	4.92
60	7.36	4.62	4.94	6.50	4.90	5.28	5.86	4.66	5.22	5.32	4.70	5.22
70	7.26	4.80	5.16	6.22	4.12	4.70	5.64	4.62	5.06	4.78	4.38	4.74
80	6.68	4.76	4.98	5.94	4.36	4.74	5.74	4.72	5.06	5.42	4.96	5.32
90	6.78	4.56	4.80	6.08	4.72	4.90	5.82	4.76	5.08	5.70	5.38	5.66
100	6.78	4.58	4.70	6.64	5.08	5.42	6.00	4.92	5.20	4.56	4.24	4.48
120	7.38	4.96	5.20	7.16	5.20	5.48	5.34	4.54	4.70	4.94	4.62	4.86
150	6.70	4.78	4.86	6.62	4.78	4.94	6.00	5.18	5.46	5.10	4.82	5.06
180	7.22	5.04	5.24	5.90	4.64	4.78	5.38	4.72	4.84	4.72	4.54	4.70
210	6.94	4.80	4.86	6.34	4.80	4.96	5.44	4.78	4.88	5.36	5.16	5.32
240	6.72	4.80	4.90	6.32	5.02	5.10	6.06	5.40	5.56	5.30	5.14	5.30
270	6.94	4.82	4.88	5.98	4.64	4.72	5.48	4.64	4.84	4.82	4.74	4.82
300	7.12	5.14	5.28	6.50	4.96	5.00	5.72	5.02	5.10	4.92	4.78	4.90
